# A Reference Hand Book of the Medical Sciences

**Published:** 1886-10

**Authors:** 


**A Reference Hand Book of the Medical Sciences.** Embracing the entire range of Scientific and Practice of Medicine and Allied Science, by various writers. Illustrated by chromo-lithographs and fine wood engravings. Edited by ALBERT H. BUCK, M. D. Vol. III. New York: Wm. Wood & Co. Buffalo: Matteson.

The third volume of this great work contains, more or less, complete references to almost every subject within the range of medicine and its collateral sciences, which alphabetically falls within the range between F A C and H Y S, and the writers of the various articles have proven the fitness of the selection for the work. It is plain that no expense is being spared in the publication of this great encyclopædia of medicine. We are informed that the number of subscribers for the work has surpassed all expectations, but the profession is always ready to show its appreciation of thoroughly good work.

